# Mode of Delivery in Drug-Dependent Pregnant Women: A Case Control Study

**DOI:** 10.1155/2017/1630967

**Published:** 2017-02-26

**Authors:** Ana Raquel Neves, Fabiane Neves, Isabel Santos Silva, Maria do Céu Almeida, Pitorra Monteiro

**Affiliations:** Department of Obstetrics B, Centro Hospitalar e Universitário de Coimbra, Coimbra, Portugal

## Abstract

*Objective*. To determine the contribution of drug use during pregnancy to the route of delivery.* Methods*. A case-control study was conducted at a hospital in Coimbra, Portugal, between 2001 and 2014. Drug-dependent pregnant women (*n* = 236) were compared with a control group of low risk women (*n* = 228) in terms of maternal characteristics, obstetric history, pregnancy complications, and labor details. Factors that influenced the mode of delivery were determined. Statistical analysis was performed with SPSS v. 23.0 (IBM Corp.).* p* values < 0.05 were considered statistically significant.* Results*. Drug-dependent women presented a lower rate of cesarean delivery (18.2 versus 28.9%, *p* = 0.006). After adjusting for the factors that were significantly related to the mode of delivery, drug dependency influenced the rate of cesarean section (*β* = 0.567; 95% CI = 0.328–0.980). Within the drug-dependent group, the mode of delivery was significantly related to previous cesarean or vaginal delivery (*p* = 0.008 and *p* < 0.001, resp.) and fetal presentation (*p* < 0.001), but not with the type of drug, route of administration, or substitution maintenance therapy.* Conclusions*. The drug-dependent group presented a significantly higher rate of vaginal delivery. However, this was not associated with the behavioral factors analyzed. We hypothesize that other social and psychological factors might explain this difference.

## 1. Introduction

Substance abuse during pregnancy is associated with considerable obstetrical morbidity and mortality. Its prevalence is largely unknown, although the USA 2013 National Survey on Drug Use and Health estimated it on 5,4% [1]. An increased risk of spontaneous abortion, preterm delivery, placental abnormalities, hypertensive complications, fetal growth restriction (FGR), low birth weight infants, neonatal abstinence syndrome, sudden infant death syndrome, and long term behavioral effects have all been associated with antenatal substance use [2–7]. However, little attention has been drawn to their effect on the mechanism of labor and delivery. In vitro studies have proved that opioids and cannabinoids exert uterorelaxant action whereas cocaine augments contractility in human myometrium [8–12]. Nevertheless, the clinical correlation of these findings has never been studied.

Our aim was to evaluate the mode of delivery and the factors that influence it in drug-dependent pregnant women.

## 2. Material and Methods

We conducted a retrospective study of drug-dependent (DD) pregnant women whose antenatal care and delivery took place at the Department of Obstetrics B of Centro Hospitalar e Universitário de Coimbra, a tertiary-care university hospital, between January 2001 and December 2014. Because the study involved completely deidentified data extraction from electronic patient records, patient consent was not required. The study was conducted according to the Declaration of Helsinki and regional authority requirements.

A total of 269 DD patients delivered in our institution during the study period. We excluded 33 women (12,3%) because they were Human Immunodeficiency Virus- (HIV-) positive (*n* = 19), had missing outcome data (*n* = 5), fetal malformations (*n* = 2), multiple pregnancy (*n* = 6), and Rh isoimmunization (one case). None of the patients reviewed presented chronic medical comorbidities other than psychiatric and/or infectious. A total of 236 DD patients were enrolled in our study. Controls were non-drug-dependent (non-DD) healthy women, randomly selected from our institutional administrative records, with a delivery date during the study period (*n* = 228).

DD patients' antenatal care was based on a multidisciplinary model involving a program of care between hospital services and drug agencies working with drug users. Antenatal care is available on a regular basis at the local drug dependency unit as well as within our institution. The control group had a model of care shared between primary care and hospital specialist services.

The patients' records were reviewed and the following data were registered:

### 2.1. Maternal Characteristics

The woman's age, educational level, ethnicity, and body mass index (BMI) were recorded. BMI was calculated upon maternal self-report of height and prepregnancy weight. Educational level was considered high if high school or higher education had been completed.

Regarding obstetric history, gravity, parity, previous cesarean section or vaginal delivery, previous abortion, and trimester of first antenatal visit were also registered.

### 2.2. Pregnancy and Neonatal Outcomes

Pregnancy complications associated with drug dependence including hypertensive disorders of pregnancy (gestational hypertension, preeclampsia, and eclampsia), placental abnormalities (placenta previa and abruptio placentae), and FGR were registered. The definitions of gestational hypertension, preeclampsia, and eclampsia were those adopted by the International Society for the Study of Hypertension in Pregnancy [13]. FGR was defined as estimated birth weight <10th centile. Delivery data included gestational age at delivery, fetal presentation at delivery, labor induction, and mode of delivery. Newborn birth weight and 5-minute Apgar score were also recorded.

### 2.3. DD Group Characteristics

A woman was considered a drug user if she had self-reported use of substitution maintenance treatment, heroin, cocaine, or cannabinoids at any time during pregnancy. Urine drug testing was performed only as an adjunct to detect or confirm suspected substance abuse after obtaining patient consent. Nonactive consumption was defined as drug withdrawal at any time during pregnancy. Data regarding the type of drug abused (opioids, cannabinoids, and cocaine), the route of administration (intravenous, smoked, and mixed), opioid replacement therapy (methadone, buprenorphine, and naloxone), associated addictions (smoking and alcohol history during the index pregnancy), and infectious diseases (HIV, Hepatitis C, Hepatitis B, and Syphilis) were recorded. HIV-positive women were excluded due to the fact that elective cesarean section was performed in all patients until 2014.

G^*∗*^Power software v. 3.1.3. was used to calculate the sample size. The mode of delivery was the endpoint used in power analysis calculation. For a power of 80% and *α* = 0.05, the sample size should be *n*1 = 368 and *n*2 = 123. After applying the exclusion criteria, we obtained a sample size of *n*1 = 355 and *n*2 = 109, with a power of 0.784 for *α* = 0.05. We accepted these values for our study.

Categorical variables were compared using the *χ*^2^ test or Fisher's exact test, according to the Cochran rules. Continuous variables were tested for normal distribution (Kolmogorov-Smirnov test). Those following a normal distribution were compared using the independent samples Student *t*-test and the independent samples Mann–Whitney *U* test was conducted to compare those that did not follow a normal distribution. Factors influencing the mode of delivery were determined using logistic regression analysis. All statistical analysis was performed using SPSS v. 23.0 (IBM Corp.). *p* values < 0.05 were considered statistically significant.

The following steps were taken to determine if drug dependency influenced the mode of delivery and the factors that influenced the mode of delivery in DD patients: the factors that predicted the mode of delivery in the study population were determined. Binary logistic regression was performed to analyze the independent influence of drug dependency in the mode of the delivery. Finally, the factors that influenced the mode of delivery within the DD group were analyzed.

## 3. Results

Maternal characteristics and pregnancy and labor details were compared with a low risk control-population of non-DD patients (Table 1). Drug-dependent patients were younger (28.40 ± 5.84 versus 29.51 ± 4.81 years, *p* = 0.021) and presented a lower BMI (21.89 ± 3.73 Kg/m^2^ versus 24.16 ± 4.27 Kg/m^2^, *p* < 0.001). The rate of nulliparity was also lower in this group (46.2% versus 64.5%, *p* < 0.001), with a higher rate of previous vaginal delivery (83.5% versus 72.8%, *p* = 0.041) despite no difference in the previous cesarean section rate. The incidence of FGR was 20.3%, significantly higher than in the control group (*p* < 0.001). Preterm delivery was also more frequent amongst drug-using women, complicating 16.6% of births (*p* < 0.001). The incidence of low birth weight was 25.8% amongst DD women compared to 13.6% in control women (*p* = 0.001). Of note is the extremely low incidence of placental abnormalities (0.8%) and hypertensive complications (3%) in the DD group.

Regarding the mode of delivery during the study period, the DD group presented a significantly lower rate of cesarean section (18.2% versus 28.9%, *p* = 0.006).

The factors that influenced the mode of delivery (vaginal delivery versus cesarean section) in the study sample were determined (Table 2). Maternal age (*p* = 0.038), nulliparity (*p* = 0.017), previous cesarean section (*p* < 0.001) or vaginal delivery (*p* < 0.001), drug dependence (*p* = 0.006), placental abnormalities (*p* = 0.001), and fetal presentation at delivery (*p* < 0.001) were significantly associated with the mode of delivery.

In order to analyze the individual effect of each of these factors on the mode of delivery, binary logistic regression analysis was conducted and demonstrated that significant independent contributions were provided by drug dependence, previous cesarean section, previous vaginal delivery, and fetal presentation (resp., *β* = 0.567, 95% CI = 0.328–0.980; *β* = 6.279, 95% CI = 3.092–12.753; *β* = 0.167, 95% CI = 0.075–0.374; and *β* = 54.689, 95% CI = 18.150–164.792; *R*^2^ = 0.416; *p* < 0.001). Among DD women, the risk of cesarean section was approximately one-half the risk among the control population.

DD group characteristics are displayed in Table 3. A total of 161 women (68.2%) presented active consumption at the time of delivery. Opioids were the most common drugs reported (89.4%). Within the 75.8% of patients under substitution maintenance therapy, methadone was the most frequent (66.1%).

Finally, the factors that influenced the mode of delivery within the DD group were determined (Table 4). No difference was found regarding the pattern of consumption. Curiously, there was no difference in relation to the mode of delivery between women with active consumption at delivery and those who stopped consumption at any time during pregnancy.

## 4. Discussion

Previous studies have focused on the influence of drug abuse on maternal and neonatal morbidity and mortality as well as on the impact of substitution maintenance therapy on these outcomes.

In view of the fact that in vitro studies have shown the influence of the analyzed drugs in myometrial function [8–12], we undertook this study to analyze the factors that influence the mode of delivery in DD pregnant women. Our results demonstrate that the DD population presented a lower rate of cesarean section. Curiously, the state of consumption, type of drug, route of administration, and substitution maintenance therapy did not contribute to this difference. This may be partially attributable to sample size as the subgroups may lack the statistical power to exclude such differences. Also, their lower BMI, obstetric history, delayed prenatal care, higher rate of coaddictions and infectious diseases, and the higher incidence of prematurity and low birth weight did not seem to influence the route of delivery.

Limitations to this study are mainly related to the fact that the study design cannot remove all potential biases and our findings might be explained by the variety of confounders presented by the study population. In particular, DD pregnant women are more likely to have a lower socioeconomical status and suffer the psychological impact of family instability and high-risk behaviors, such as prostitution and violence. These factors, associated with negative feelings about pregnancy or an unintended or unwanted pregnancy, may contribute to delayed medical care during labor, with patients attending the hospital at a more advanced stage of labor. Therefore, we hypothesize that psychological factors may explain the difference in the route of delivery in DD pregnant women demonstrated in this study.

Further research is needed to elucidate the underlying psychosocial reasons for the higher rate of vaginal delivery found in our study.

## Figures and Tables

**Table 1:** Maternal characteristics and pregnancy and neonatal outcomes of the study groups.

	DD group (*n* = 236)	Non-DD group (*n* = 228)	Odds ratio (95% CI)	*p* value
*Maternal characteristics*				
Maternal age (years)	28.40 (5.84)	29.51 (4.81)		0.021
Body mass index (Kg/m^2^)	21.89 (3.73)	24.16 (4.27)		<0.001
Ethnicity			1.463 (0.458–4.679)	0.519
Caucasian	231 (97.9)	221 (96.9)		
Educational level			0.130 (0.084–0.202)	<0.001
Low	170 (77.6)	60 (31.1)		
Nulliparous	109 (46.2)	147 (64.5)	0.473 (0.326–0.687)	<0.001
Within multiparous				
Previous cesarean section	22 (17.3)	22 (27.2)	0.562 (0.287–1.100)	0.090
Previous vaginal delivery	106 (83.5)	59 (72.8)	2.002 (1.022–3.922)	0.041
*Pregnancy complications*				
Placental abnormalities	2 (0.8)	3 (1.8)	0.641 (0.106–3.872)	0.625
Hypertensive complications	7 (3.0)	5 (2.2)	1.363 (0.426–4.359)	0.600
Fetal growth restriction	48 (20.3)	6 (2.6)	9.447 (3.955–22.563)	<0.001
*Labor and neonatal outcomes*				
Gestational age (weeks)	37.97 (1.53)	38.83 (31.20)		<0.001
Prematurity (<37 weeks)	39 (16.6)	9 (3.9)	4.842 (2.287–10.250)	<0.001
32^+0^–36^+6^ weeks	35 (14.9)	9 (3.9)	4.258 (1.997–9.080)	<0.001
28^+0^–31^+6^ weeks	4 (1.7)	0	0.503 (0.460–0.551)	0.124
Fetal presentation			0,963 (0.496–1.870)	0.912
Cephalic	217 (91.9)	209 (91.7)		
Noncephalic	19 (8.1)	19 (8.3)		
Induction of labor	58 (24.9)	60 (26.3)	0.928 (0.611–1.410)	0.726
Cesarean section	43 (18.2)	66 (28.9)	0.547 (0.353–0.847)	0.006
Newborn birth weight (g)	2812.23 (456.59)	3180.47 (2812.23)		<0.001
<2500 g	61 (25.8)	31 (13.6)	2.215 (1.374–3.572)	0.001
<1500 g	4 (1.7)	0	0.504 (0.460–0.552)	0.124
5 min Apgar score <7	2 (0.8)	0	0.506 (0.463–0.554)	0.499

Values are mean (standard deviation) for continuous variables or *n* (%) for categorical variables.

**Table 2:** Factors that influenced the mode of delivery in the study population.

	Vaginal delivery (*n* = 355)	Cesarean section (*n* = 109)	Odds ratio (95% CI)	*p* value
*Maternal characteristics*				
Maternal age (years)	28.75 (5.08)	29.88 (5.22)		0.038
Body mass index (Kg/m^2^)	22.45 (4.09)	23.86 (4.45)		0.055
Ethnicity			0.919 (0.244–3.456)	0.901
Caucasian	346 (97.5)	106 (97.2)		
Non-Caucasian	9 (2.5)	3 (2.8)		
Nulliparity	185 (52.1)	71 (65.1)	1.717 (1.100–2.681)	0.017
Previous cesarean section	18 (5.1)	26 (23.9)	5.865 (3.070–11.203)	<0.001
Previous vaginal delivery	153 (43.1)	12 (11.0)	0.163 (0.087–0.301)	<0.001
Drug dependence	193 (54.5)	43 (39.1)	0.547 (0.353–0.847)	0.006
*Pregnancy complications*				
Placental abnormalities	0	5 (4.6)	0.227 (0.191–0.260)	0.001
Hypertensive complications	10 (2.8)	2 (1.8)	0.645 (0.139–2.989)	0.572
Fetal growth restriction	44 (12.4)	10 (12.2)	0.714 (0.397–1.471)	0.359
*Labor characteristics*				
Gestational age (weeks)	38.43 (1.41)	38.48 (1.47)		0.581
Fetal presentation			30.395 (11.491–80.400)	<0.001
Cephalic	350 (98.6)	76 (69.7)		
Noncephalic	5 (1.4)	33 (30.3)		
Induction of labor	95 (27.0)	23 (21.1)	0.724 (0.432–1.213)	0.218

Values are mean (standard deviation) for continuous variables or *n* (%) for categorical variables.

**Table 3:** Drug dependent group characteristics.

	*n* = 236
*Pattern of consumption*	
Active consumption during pregnancy	161 (68.2)
Type of drug	
Cocaine	99 (41.9)
Opioids	211 (89.4)
Cannabinoids	69 (29.2)
Mixed	123 (52.1)
Only opioids	93 (39.4)
Only cannabinoids	16 (6.8)
Only cocaine	4 (1.7)
Route of administration	
Smoked	113 (47.9)
Intravenous	41 (17.4)
Mixed	22 (9.3)
*Substitution maintenance therapy*	
Methadone	156 (66.1)
Buprenorphine	18 (7.6)
Naltrexone	5 (2.1)
None	57 (24.2)
*Comorbidities*	
Psychiatric disease	58 (24.6)
Infectious diseases	
Hepatitis C	118 (50)
Hepatitis B	6 (2.5)
Syphilis	6 (2.5)
*Coaddictions*	
Smoking history	180 (76.3)
Alcohol abuse	15 (6.4)

Values are *n* (%).

**Table 4:** Factors that influence the mode of delivery in drug-dependent patients.

	Vaginal delivery (*n* = 193)	Cesarean section (*n* = 43)	Odds ratio (95% CI)	*p* value
*Maternal characteristics*				
Maternal age (years)	28.13 (5.28)	29.39 (5.86)		0.297
Body mass index (Kg/m^2^)	21.82 (3.85)	22.04 (3.28)		0.452
Ethnicity			0.889 (0.097–8.157)	0.917
Caucasian	189 (97.9)	42 (97.7)		
Non-Caucasian	4 (2.1)	1 (2.3)		
Nulliparous	84 (43.5)	25 (58.1)	1.802 (0.923–3.520)	0.082
Previous cesarean section	13 (6.7)	9 (20.9)	3.665 (1.453–9.248)	0.008
Previous vaginal delivery	97 (50.3)	9 (20.9)	0.262 (0.119–0.576)	<0.001
*Pattern of consumption*				
State of consumption			0.646 (0.325–1.281)	0.209
Active	135 (70.3)	26 (60.5)		
Nonactive	57 (29.7)	17 (39.5)		
Type of drug				
Only opioids	71 (36.8)	22 (51.2)	1.800 (0.925–3.503)	0.081
Only cocaine	3 (1.6)	1 (2.3)	1.508 (0.153–14.857)	0.555
Only cannabinoids	15 (7.8)	1 (2.3)	0.283 (0.036–2.199)	0.317
Mixed	104 (53.9)	19 (44.2)	0.677 (0.348–1.317)	0.350
Route of administration				
Intravenous	33 (17.2)	8 (18.6)	1.101 (0.469–2.589)	0.825
Smoked	91 (47.4)	22 (51.2)	1.163 (0.600–2.253)	0.655
Mixed	17 (77.3)	5 (22.7)	1.354 (0.471–3.898)	0.566
Substitution maintenance therapy				
Methadone	128 (66.3)	28 (65.1)	0.948 (0.473–1.898)	0.880
Buprenorphine	13 (6.7)	5 (11.6)	1.822 (0.613–5.414)	0.336
Naltrexone	4 (2.1)	1 (2.3)	1.125 (0.123–10.324)	1.000
None	48 (24.9)	9 (20.9)	0.800 (0.358–1.787)	0.585
*Infectious diseases*				
Hepatitis C	91 (47.2)	27 (62.8)	1.891 (0.958–3.733)	0.064
Hepatitis B	4 (2.1)	2 (4.7)	2.305 (0.408–13.009)	0.300
Syphilis	4 (2.1)	2 (4.7)	2.305 (0.408–13.009)	0.300
*Associated addictions*				
Smoking history	148 (82.2)	32 (78)	0.769 (0.334–1.767)	0.535
Alcohol use	12 (6.8)	3 (7.5)	1,115 (0.299–4.150)	0.871
*Pregnancy data*				
Delayed prenatal care	123 (63.7)	29 (67.4)	1.179 (0.584–2.379)	0.646
Placental abnormalities	0	2 (4.7)	0.175 (0.133–0.231)	0.033
Hypertensive complications	5 (2.6)	2 (4.7)	1.834 (0.344–9.785)	0.614
Fetal growth restriction	39 (20.2)	9 (20.9)	1.045 (0.463–2.360)	0.915
*Labor characteristics*				
Gestational age (weeks)	37.98 (1.56)	37.86 (1.73)		0.895
Fetal presentation			25.313 (7.839–81.734)	<0.001
Cephalic	189 (97.9)	28 (65.1)		
Noncephalic	4 (2.1)	15 (34.9)		
Induction of labor	49 (25.8)	9 (20.9)	0.762 (0.341–1.701)	0.506
Newborn birth weight (g)	2781.99 (508.96)	2719.30 (541.49)		0.786

Values are mean (standard deviation) for continuous variables or *n* (%) for categorical variables.

## Abbreviations

BMI: Body mass index
CI: Confidence interval
DD: Drug dependent
FGR: Fetal growth restriction
HIV: Human Immunodeficiency Virus.
